# Highly specific C–C bond cleavage induced FRET fluorescence for *in vivo* biological nitric oxide imaging†
†Electronic supplementary information (ESI) available. See DOI: 10.1039/c6sc04071c



**DOI:** 10.1039/c6sc04071c

**Published:** 2016-11-30

**Authors:** Hua Li, Deliang Zhang, Mengna Gao, Lumei Huang, Longguang Tang, Zijing Li, Xiaoyuan Chen, Xianzhong Zhang

**Affiliations:** a Center for Molecular Imaging and Translational Medicine , State Key Laboratory of Molecular Vaccinology and Molecular Diagnostics , School of Public Health , Xiamen University , 361102 Xiamen , Fujian , China . Email: zhangxzh@xmu.edu.cn ; Email: zijing.li@xmu.edu.cn; b Laboratory of Molecular Imaging and Nanomedicine (LOMIN) , National Institute of Biomedical Imaging and Bioengineering (NIBIB) , National Institutes of Health (USA) , Bethesda , Maryland 20892 , USA

## Abstract

A novel Förster resonance energy transfer (FRET) fluorescence “off–on” system based on the highly specific, sensitive and effective C–C bond cleavage of certain dihydropyridine derivatives was reported for real-time quantitative imaging of nitric oxide (NO). 1,4-Dihydropyridine was synthesized as a novel linker which could connect customized fluorophores and their corresponding quenchers. The specific and quantitative response to NO is confirmed using fluorescence spectrometry with the classical example of fluorescein isothiocyanate (FITC) and [4′-(*N*,*N*′-dimethylamino)phenylazo] benzoyl (DABCYL). The fluorescence intensity increased linearly with the increase in the amount of NO. Cells incubated with an exogenous NO donor emitted fluorescence as expected. A high fluorescence intensity was detected in macrophages which generate NO when incubated with lipopolysaccharide (LPS). The *in vivo* imaging shows about an 8-fold contrast between Freund's adjuvant stimulated feet and normal feet in mice after intravenous injection, which was the first example of *in vivo* semiquantitative fluorescence imaging of NO in mammals.

## Abstract 2

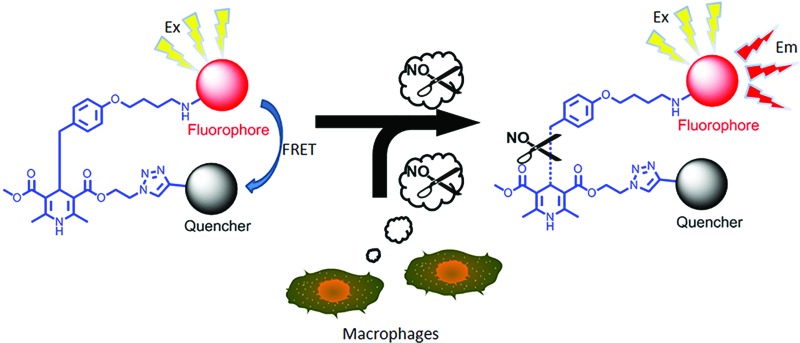
A novel FRET fluorescence “off–on” system based on the highly specific, sensitive and effective C–C bond cleavage of certain dihydropyridine derivatives was reported for real-time quantitative imaging of nitric oxide (NO).

## Introduction

Nitric oxide (NO), a very active radical signaling molecule, has been found to exist and play a significant role in living body pathophysiology.^1^ It has been gradually realized that NO is involved in different physiological processes including vasodilation, wound healing, immune responses and nerve cell communication.^2^ Therefore, NO detection *in vitro* or, more ideally, 3D imaging the distribution and concentration of NO *in vivo* would be of a great help in understanding the metabolism of NO.

Various methods, such as electrochemistry,^3^ optical imaging (fluorescence, chemiluminescence and bioluminescence imaging),^4^ electron paramagnetic resonance (EPR),^5^ and magnetic resonance (MR), have been developed to image NO.^6^ Among these methods, optical probe-based techniques are the most available for NO imaging. With intrinsic molecular sensitivity, high resolution, repeatability, a high level of safety, and a relatively low instrumentation cost, optical probes have become important tools in the study of cells and small animals. Both fluorescence and chemiluminescence imaging have been widely used for elucidating the function of NO in different biological systems.^7^

Among the existing ratiometric probes, most of them are designed according to a photo-induced electron transfer (PET) mechanism, during which donor–π–acceptor structures are formed after the reaction between NO and the probe. Numerous efforts have been made on *o*-diamino aromatic compounds which could be oxidized by NO into triazole derivatives in the presence of oxygen.^8^ Although fluorescein based probes have been effective in zebrafish,^9^ there are no results regarding mammalian models yet. The speculated reason is the deficiency of oxygen at site for NO detection. Furthermore, *o*-diamino aromatics are easily disturbed by some oxidative species secreted in cells, such as H_2_O_2_ and OONO^–^.^10^ A series of copper complexes were tested in mouse models, which could obviously distinguish a normal liver from a liver with acute severe hepatic injury (ASHI).^11^ Yet, the fluorescence images were obtained from excised liver slices harvested from a living animal using an *ex vivo* method. There are other limitations of PET probes:^12^ (1) the measured excitation and emission wavelengths are often different from the expected values; (2) relatively broad fluorescence spectra are often detected for PET probes; (3) although remarkable advances have been made in computational chemistry, the quantum yield of fluorophores cannot be accurately predicted.

On the other hand, FRET mechanism based fluorescent probes have been used in the fields of chemistry and biology for decades. The commercially available FRET fluorophores have been proven to have certain excitation and emission wavelengths and an exact quantum yield. With different fluorophores, the available detection range could be extended to the near-infrared region, which makes FRET probes more promising for *in vivo* imaging than PET probes. A typical FRET quencher–fluorophore pair is FITC and DABCYL. This pair can directly indicate if the bonds between the two moieties are broken. A probe based on coumarin decorated 1,4-dihydropyridine was synthesized and evaluated *in vitro*, which was designed based on stoichiometry.^13^ The probe transforms into a conjugated fluorophore after NO stimulation, but the *in vivo* application was largely restricted by the short emission wavelength. Therefore, we designed and synthesized a FRET based probe to conquer the previous obstacles.

**Scheme 1:**
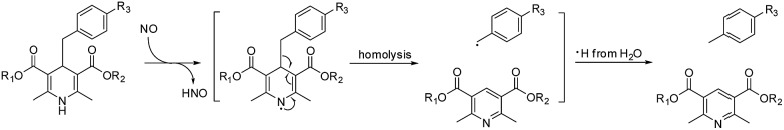
The most acceptable mechanism for the reaction between 1,4-dihydropyridine and NO.

Herein, we present a fluorophore–quencher FRET “off–on” system for detecting and imaging NO *in vivo* in a non-invasive and real-time way. The 1,4-dihydropyridine derivatives can react with NO specifically. Furthermore, as the C4-position is substituted with a benzyl group, the C–C bond between 1,4-dihydropyridine and the benzyl group can be cleaved through NO stimulation with a high yield (Scheme 1).^14^ The reaction is very specific and sensitive.

## Results and discussion

**Scheme 2:**
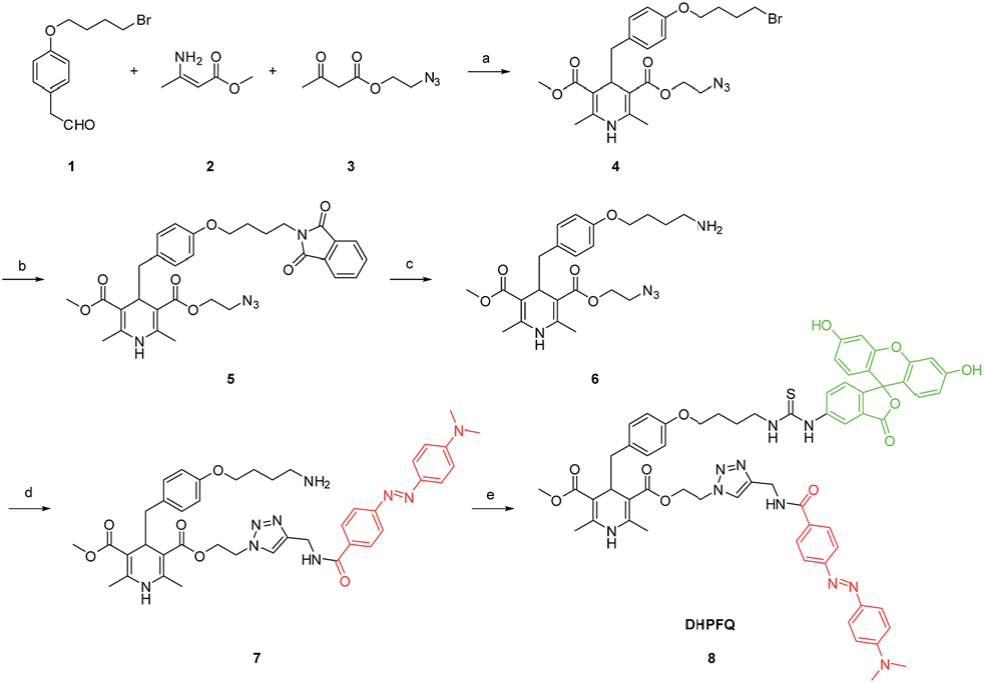
Synthesis of **DHPFQ**. Reagents and conditions: (a) EtOH, reflux; (b) DMF, potassium phthalimide, rt; (c) EtOH, N_2_H_4_·H_2_O, rt; (d) THF, DABCYL-yne, CuI, DIPEA, rt; (e) DMF, FITC, rt.

In our probes, FITC and DABCYL were linked *via* 1,4-dihydropyridine within a distance of 10 nm. Because of the FRET mechanism, DABCYL quenches the fluorescence emitted by FITC in the absence of NO. The cleavage of the C–C bond leads to the release of DABCYL and the fluorescence can be detected again (Fig. S4†). To achieve an image *in vivo*, the emission wavelength is tuned to increase the signal–noise ratio through using different fluorophores (Scheme 2).

To obtain the NO target probe, an asymmetric synthesis strategy was used in the Hanztzsch reaction. An equivalent amount of aldehyde, β-keto ester and methyl 3-aminobut-2-enoate ester were combined into an asymmetrical 1,4-dihydropyridine (compound **4**) which had only one azido moiety.^15^ Compound **6** was designed as a linker (Scheme 2). The amino group on compound **6** is the binding site for the fluorophore, because many commercial fluorophores are designed for labeling the amino groups on peptides and proteins. The azido group would react with an yne group which was linked to a quencher. DABCYL-yne and FITC were selected to react with the linker to obtain the target probe. The reaction between **DHPFQ** (dihydropyridine–fluorophore–quencher) and NO was evaluated in phosphate buffer using a fluorospectrometer, and the reaction proceeded in a quantitative way. The C–C bond cleavage was confirmed from the increase in the intensity of the fluorescence.

**Fig. 1:**
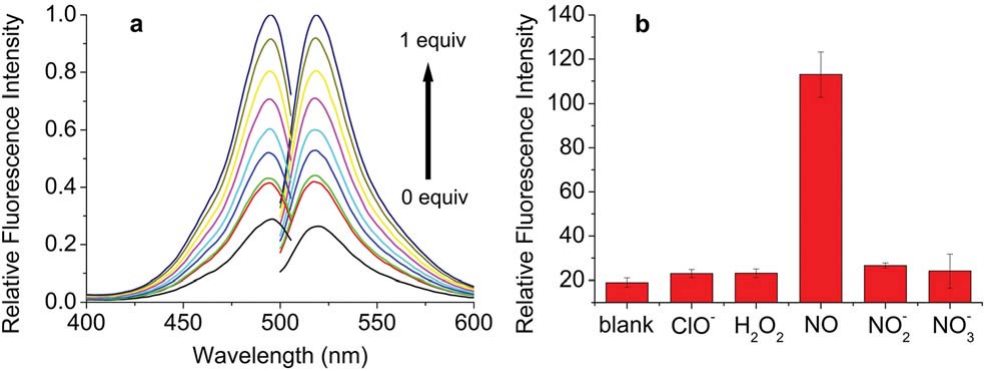
(a) Fluorescence response of 10 μM **DHPFQ** to 1 equivalent of NO in CH_3_CN/PBS buffer = 1/4 (pH 7.4, *λ*_ex_ = 490 nm, *λ*_em_ = 525 nm); (b) 10 μM **DHPFQ** was exposed to the same equivalence of NO and other ROS/RNS..

As expected, an enhancement of the fluorescence intensity at certain excitation (490 nm) and emission (525 nm) wavelengths was detected in 2 min after **DHPFQ** reacted with NO (Fig. 1a). The fluorescence intensity increases with an increasing amount of NO (diluted NO saturated solution).^16^

Most small molecule inflammatory factors in the complicated internal environment are reactive oxygen/nitrogen species (ROS/RNS). Therefore, specific recognition and reaction between the probes and NO is essential for *in vivo* applications. To evaluate the reaction selectivity, compound **S** (Scheme S2†) as a **DHPFQ** analog was treated with different reactive species firstly (Fig. S1†). About 98% of compound **S** was found to be decomposed when treated with NO, which was measured using HPLC. Furthermore, **DHPFQ** was treated with NO and many other reactive species (Fig. 1b). The decomposition was measured using a fluorescence spectrometer. **DHPFQ** showed a significant increase in fluorescence intensity with NO. No significant decomposition was found when **DHPFQ** and compound **S** were treated with other reactive species. The results reveal the exclusive response of 1,4-dihydropyridine derivatives to no ROS or RNS but NO. The UV spectra of **DHPFQ** before and after NO treatment were also investigated, which showed that activated and inactivated **DHPFQ** share similar profiles (Fig. S3†).

With excellent responsiveness to NO and exceptional selectivity *in vitro* confirmed, the ability of **DHPFQ** to visualize NO in live cells was further tested. Sodium nitroprussiate (SNP) as an exogenous NO donor was used to validate the high contrast. Compared to the control group (Fig. S7a†), the SNP treated group (Fig. S7b†) showed strong fluorescence, which implies that **DHPFQ** could respond to exogenous NO in a physiological environment.

**Fig. 2:**
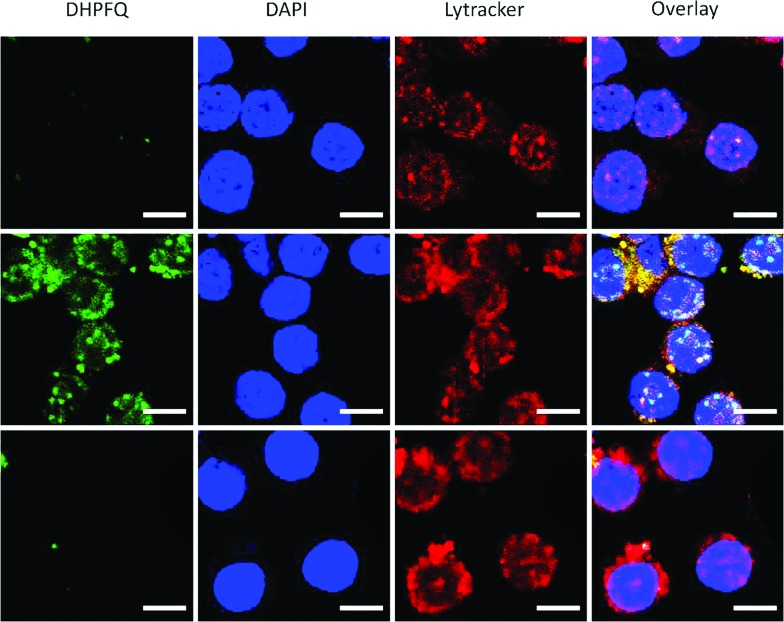
**DHPFQ** detection of NO in Raw 264.7 cells. (Top) Incubated with **DHPFQ** (50 μM) for 8 h; (Middle) pretreated with LPS (0.5 μg mL^–1^) for 4 h and incubated with **DHPFQ** (50 μM) for 8 h; (Bottom) pretreated with *N* ^G^-monomethyl-l-arginine (2 mM), a NOS inhibitor, for 1.5 h and incubated with LPS (0.5 μg mL^–1^) for 4 h, then **DHPFQ** (50 μM) for 8 h. Green, **DHPFQ**; blue, DAPI; red, Lytracker Red. Scale bars are all 10 μm.

Since NO is an inflammatory factor generated by macrophages when stimulated with an antigen, in order to test the feasibility of application *in vitro*, raw 264.7 cells were stimulated with lipopolysaccharide (LPS) to generate endogenous NO.^17^ A significant enhancement of the fluorescence intensity was observed when the macrophages were incubated with **DHPFQ** and LPS (Fig. 2, middle). There was a high correspondence between the green fluorescence and the Lytracker Red signal which suggests this probe is metabolized in lysosomes. In contrast, when the macrophages were pretreated with *N* ^G^-monomethyl-l-arginine (l-NMA), an inhibitor of nitric oxide synthase (NOS),^18^ only a weak fluorescence signal above the background was detected (Fig. 2, bottom). This result demonstrated that the fluorescence is indeed from the switch-on reaction between **DHPFQ** and NO. **DHPFQ** has the ability to detect endogenous NO *in vitro*, suppress interference from other inflammatory factors, and has the potential to detect NO *in vivo*.

**Fig. 3:**
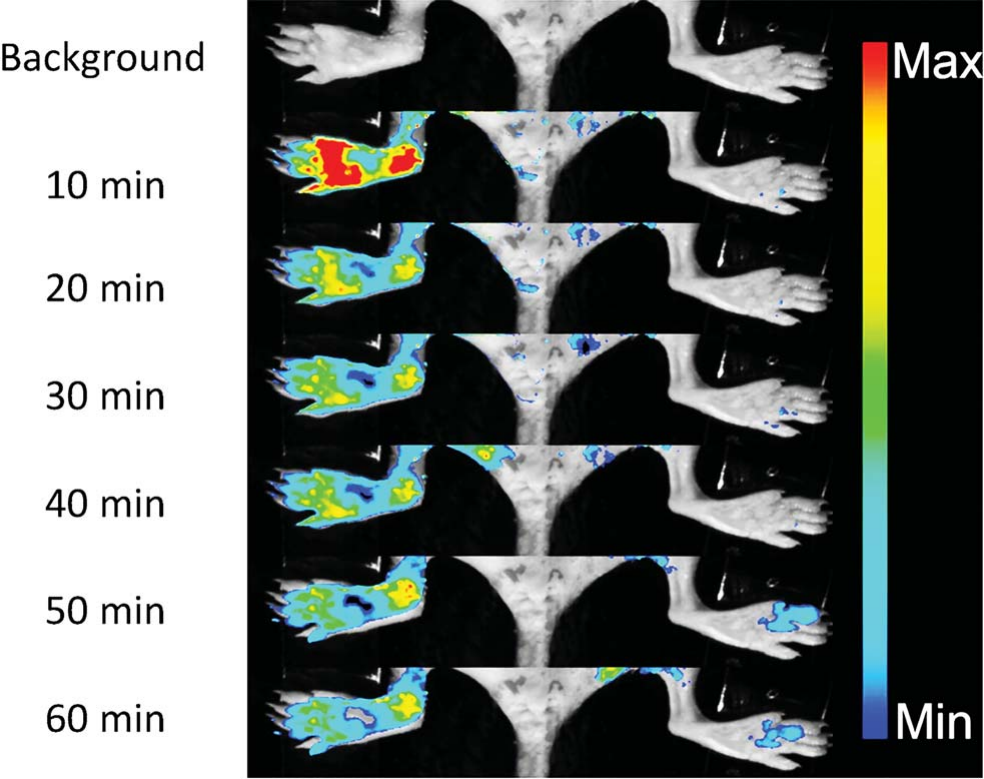
*In vivo* fluorescence images of an NO rich inflamed foot (left) and the control foot (right) before and after the iv injection of **DHPFQ** (0.5 mg kg^–1^).

NO, as a significant inflammatory factor, is concentrated in inflamed areas.^19^ An inflamed mouse model was then examined to test whether **DHPFQ** is effective *in vivo*. Freund's adjuvant was subcutaneously injected into the left rear paws of mice to trigger inflammation.^20^ After two weeks, another portion of Freund's adjuvant was subcutaneously injected. At 3 days after the second injection, **DHPFQ** was intravenously injected (injected dose: 0.5 mg kg^–1^, 10% ethanol, 90% physiological saline, 100 μL) and the mice were imaged after 10 min to 60 min using an *in vivo* imaging system (Fig. 3). To reduce the background absorption, depilatory paste was used to partially remove hair from the mice. 470 nm and 600 nm were chosen as the excitation and emission wavelengths to improve the signal to noise ratio. The fluorescence intensity observed for the inflamed region was about 8-times higher than that of the normal area in the first 10 min after injection.

**Fig. 4:**
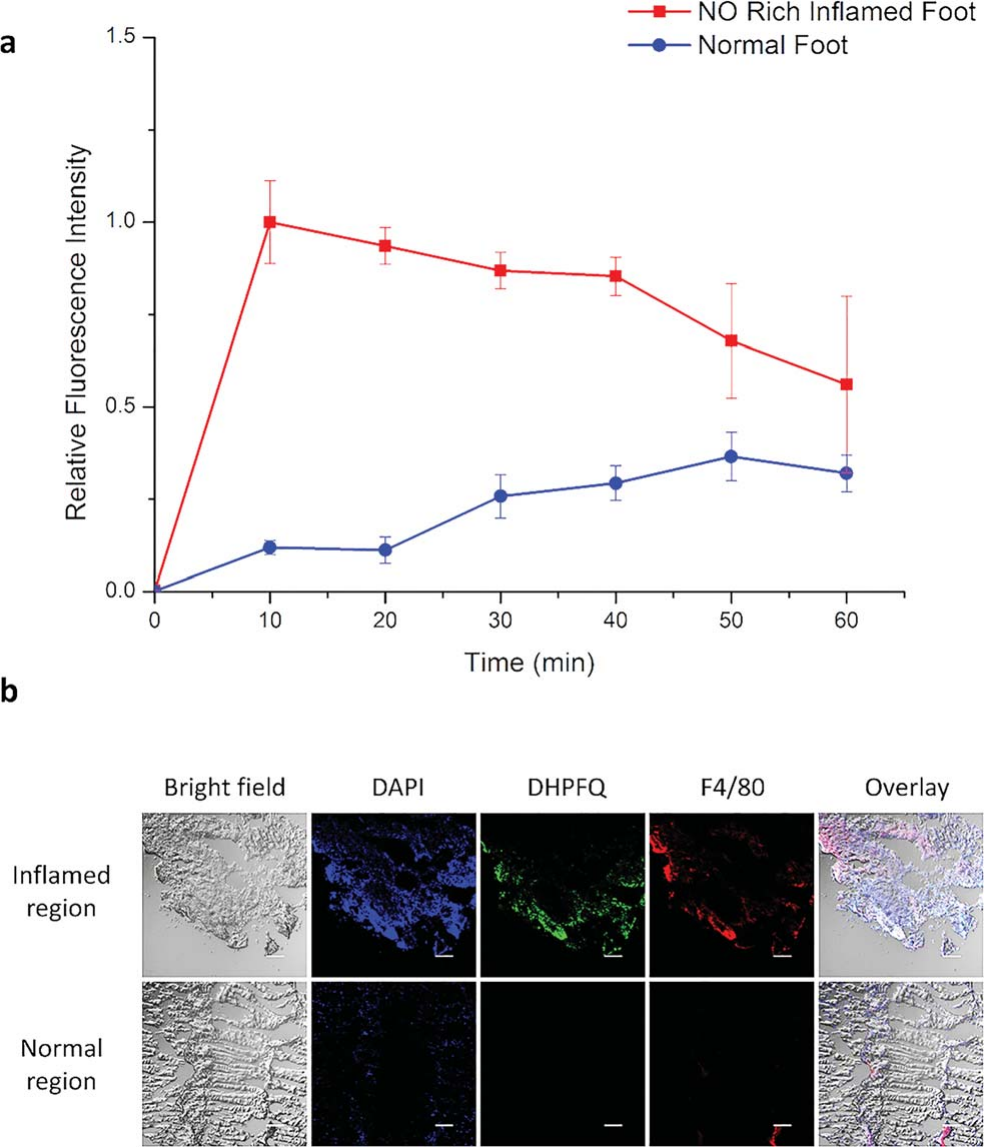
(a) The relative fluorescence signal for an NO rich inflamed foot and a normal foot after iv injection. (b) Immunohistology staining of excised muscle slices harvested from live animals at 10 min after the injection of **DHPFQ**. Scale bars are all 50 μm. Blue, DAPI; green, **DHPFQ**; red, F4/80.

Inflammation region kinetics and normal region curves were obtained using semiquantitative analysis of the fluorescence image, which was analyzed by selecting the region of interest (ROI) (Fig. 4a). After iv injection, **DHPFQ** was switched on immediately in the inflamed region. The fluorescence intensity was about 8-times higher in the inflamed region over the normal region within the first 10 min post-injection. After 60 min, about 2-fold fluorescence intensity was observed in the inflamed region compared to the normal region.

The immunohistology staining (Fig. 4b) also indicated that NO was generated within inflammatory tissues where macrophages were crowded (which is indicated from F4/80 staining) and the NO concentration was significantly higher than in the normal region. The concentration of **DHPFQ** decreased with time, which diminished the fluorescence intensity of the inflamed tissue. Because of the metabolism, switch-on **DHPFQ** can be transported to normal tissue through blood circulation, leading to a fluorescence signal at normal organs as well. Some studies suggested that the concentration of NO in physiological environments ranges from 100 pM up to 5 nM,^21^ which indicates that **DHPFQ** has a lower limit of detection at the nM level.

The comparison of existing imaging methods and the probe developed in this study is summarized in Table 1. **DHPFQ** possesses the advantages of optical imaging, and low specificity can be overcome with the application of the 1,4-dihydropyridine structure. In this study, FITC and DABCYL were used to demonstrate the feasibility of the fluorescence imaging of NO *in vivo*. NIR fluorophores and quenchers could substitute FITC and DABCYL to obtain a FRET probe with good penetration.

**Table 1:** Comparison of molecular imaging methods and probes for NO detection

Modality	Resolution	Specificity	Sensitivity	Penetration	Ref.
EPR	mm	High	nM	Good	5 and 22
MRI	mm	High	μM	Good	6*b*
Optical	μm	Low	nM	Medium	8*b* and 23
**DHPFQ**	μm	High	nM	Good (potential)	This work

## Conclusions

A novel C–C bond cleavage induced FRET fluorescent probe **DHPFQ** based on 1,4-dihydro-pyridine was synthesized. *In vitro* assessments have shown a ratiometric and specific result in responding to NO over other RNS/ROS. The fluorescence intensity increased linearly with the increasing amount of NO. Cell assay also supports the specificity. With improved specificity, **DHPFQ** is the first fluorescent probe applied in mammals. Furthermore, semiquantitative results were obtained through ROI analysis. The penetration property of NIR probes is tunable simply through the substitution of the fluorophores and quenchers, which will make the probe more practical for clinical applications.
