# Supplementary material for: Highly specific C–C bond cleavage induced FRET fluorescence for in vivo biological nitric oxide imaging
Source: Chem Sci. 2016 Nov 30;8(3):2199–203. doi: 10.1039/c6sc04071c (PMC5407267; doi:10.1039/c6sc04071c)
Supplement: Supplementary file 1 [file SC-008-C6SC04071C-s001.pdf]

Electronic Supplementary Material (ESI) for Chemical Science.
This journal is © The Royal Society of Chemistry 2016

Supplementary Information

Highly Specific C-C Bond Cleavage Induced FRET Fluorescence for *in vivo* Biological Nitric Oxide Imaging

Hua Li,[a] Deliang Zhang,[a] Mengna Gao,[a] Lumei Huang, [a] Longguang Tang, [a] Zijing Li*,[a] Xiaoyuan Chen[b] and Xianzhong Zhang*[a]

[a]Center for Molecular Imaging and Translational Medicine, State Key Laboratory of Molecular Vaccinology and Molecular Diagnostics, School of Public Health, Xiamen University, 361102 Xiamen, Fujian, China

[b]Laboratory of Molecular Imaging and Nanomedicine (LOMIN), National Institute of Biomedical Imaging and Bioengineering (NIBIB), National Institutes of Health (USA). Bethesda, Maryland 20892, United States

TABLE OF CONTENT

MATERIALS AND METHODS 2

EXPERIMENTS AND CHARACTERIZATIONS 3

SPECIFICITY ASSAY ON COMPOUND S IN AQUEOUS SOLUTION 9

CORRELATION BETWEEN NO AMOUNT AND FLUORESCENCE INTENSITY 10

UV SPECTRA ASSAY 11

IDENTIFICATION OF THE PRODUCTS OF DHPFQ TREATED WITH NO 11

CYTOTOXICITY OF DHPFQ 12

FLUORESCENCE IMAGING ON FLUORESCENCE MICROSCOPE 14

ANIMAL MODELS 15

IMMUNOHISTOLOGY STAINING 15

IN VIVO FLUORESCENCE IMAGING 15

APPENDIX 16

REFERENCES 21

Materials and methods

Reagents and solvents were purchased from commercial resources and used without further purification. The ^{1}H NMR spectra and ^{13}C NMR were obtained on a Bruker spectrometer (Avance III 600 MHz). Mass spectrometry was acquired from a Bruker Equire 3000 plus (ESI) instrument. High-resolution mass spectrometry was acquired from a Q Exactive LC-MS/MS system. HPLC analysis was achieved on a HYPERSIL GOLD C18 coloum (Thermo Scientific, 5 μm, 10 mm × 250 mm). Fluorescence spectra were recorded by a Perkin Elmer LS55 fluorescence spectrophotometer. Fluorescence images of cells were collected on a Nikon Ti-U inverted fluorescence microscope. *In vivo* imaging was performed on a Kodak Carestream Fx Pro/FX. Confocal microscopy imaging was acquired from a Olympus ix83 system.

Experiments and characterizations

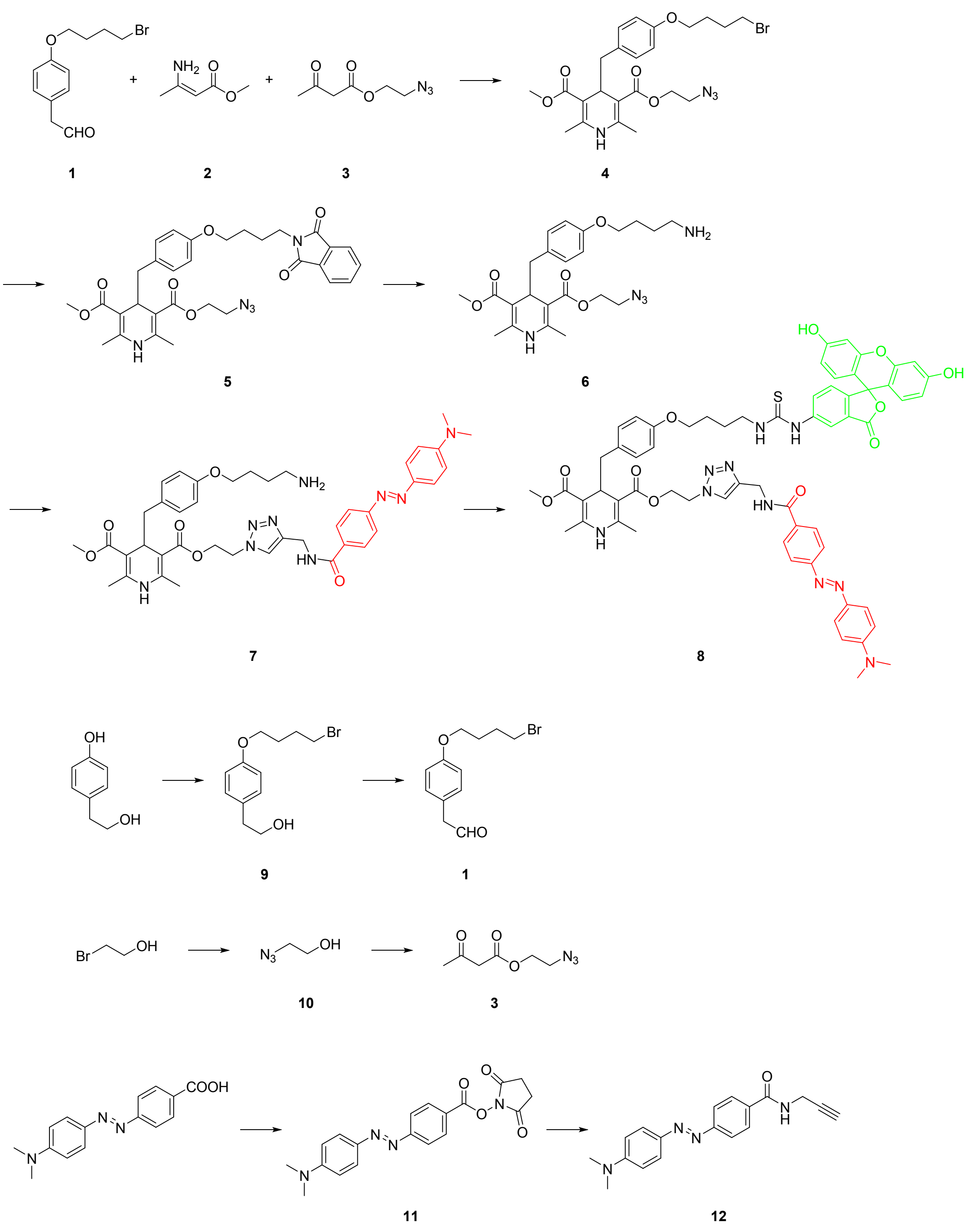

Scheme S1. Synthetic scheme of **DHPFQ**.

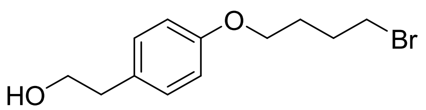

2-(4-(4-Bromobutoxy)phenyl)ethanol (9)

4-(2-Hydroxyethyl)phenol (2.76 g, 20 mmol), 1,4-dibromobutane (4.75 g, 22 mmol), KI (1.00 g, 6 mmol) and K_2CO_3 (13.80 g, 100 mmol) were dissolved in CH_3CN (100 mL) and stirred for 36 h at room temperature. The mixture was filtered and the filtrate was evaporated in vacuo. The residue was purified by column chromatography (silica gel, petroleum ether/ ethyl acetate 3:1) to give a white solid (3.33 g,12 mmol, 61%). ^{1}H NMR ($CDCl_3$, 400 MHz) δ: 6.91 (2H, d, J = 4.0 Hz), 6.72 (2H, d, J = 4.0 Hz), 5.22 (1H, s), 4.15 (1H, t, J = 4.8 Hz), 3.96 (2H, t, J = 4.8 Hz), 3.65 (6H, s), 3.50 (2H, t, J = 6.4 Hz), 2.52 (2H, d, J = 4.8 Hz), 2.18 (6H, s), 2.09-2.02 (2H, m), 1.95-1.92 (2H, m). ^{13}C NMR ($CDCl_3$, 100 MHz) δ: 168.3, 145.6, 141.1, 130.8, 114.5, 113.3, 101.5, 66.9, 50.9, 41.2, 35.4, 33.6, 24.9, 19.2. HRMS (FTMS ESI$^+$): m/z calcd for $C_{22}H_{28}BrNNaO_5^+$ 488.1044 [M + H]$^+$; found 488.1049.

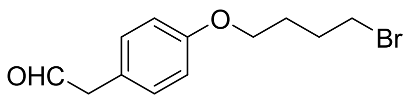

2-(4-(4-Bromobutoxy)phenyl)acetaldehyde (1)

9 (1.36 g, 5.0 mmol) and 4Å molecular sieve (1 g) in CH_2Cl_2 (100 mL) was stirred in an ice/ water bath. Pyridine chlorochromate (2.16 g, 10 mmol) was added in 30 min. The mixture was stirred at 0 ^{o}C for 3 h and filtered through a thin silica gel pad. The filtrate was evaporated in vacuo to give a yellow oil (0.61 g, 2.2 mmol, 72%).

Compound **2, 3, 11** were synthesized following a previous literature.[1-3]

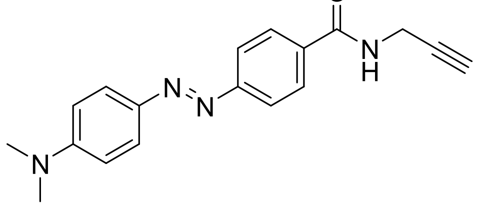

4-((4-(Dimethylamino)phenyl)diazenyl)-N-(prop-2-ynyl)benzamide (12)

A solution of compound **11** (0.37 g, 1 mmol), prop-2-yn-1-amine (0.11 g, 2 mmol) and N-ethyl-N-isopropylpropan-2-amine (DIPEA, 0.1 mL) completely dissolved in DMF was stirred for 24 hours. The mixture was evaporated in vacuo. The residue was purified by column chromatography (silica gel, CH_2Cl_2/ ethyl acetate from 100:0 to 100:5) to yield a red solid (0.20 g, 0.65 mmol, 65%). ^{1}H NMR δ 7.95-7.88 (m, 6H), 6.78 (d, 2H, J = 9.2 Hz), 6.33 (t, 1H, J = 4.3 Hz), 4.31 (dd, 2H, J_1 = 4.3 Hz, J_2 = 2.6 Hz), 3.14 (s, 6H), 2.33 (t, 1H, J = 2.6 Hz). ^{13}C NMR δ 166.63, 155.32, 152.88, 127.93, 125.47, 122.32, 111.48, 72.05, 40.31, 29.89, 24.77. HRMS (FTMS ESI+): m/z calcd for $C_{18}H_{18}N_4O^+$ 307.1559 $[M + H]^+$; found 307.1554.

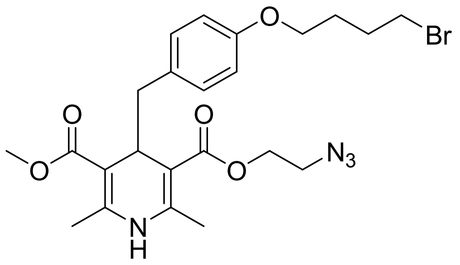

3-(2-Azidoethyl) 5-methyl 4-(4-(4-bromobutoxy)benzyl)-2,6-dimethyl-1,4-dihydropyridine- 3,5-di-carboxylate (4)

A mixture of **1** (0.68 g, 2.5 mmol), **2** (0.29 g, 2.5 mmol) and **3** (0.42 g, 2.5 mmol) in ethanol (50 mL) was stirred for 2.5 h under reflux condition. The solvent was evaporated in vacuo to give the residue which was purified by column chromatography (silica gel, petroleum ether/ ethyl acetate 3:1) to give the yellow solid (0.53 g, 12 mmol, 41%).

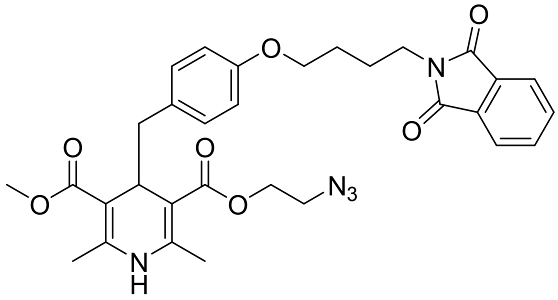

3-(2-Azidoethyl) 5-methyl 4-(4-(4-(1,3-dioxoisoindolin-2-yl)butoxy)benzyl)-2,6- dimethyl-1,4-dihydropyridine-3,5-dicarboxylate (5)

A solution of **4** (1.54 g, 3 mmol), potassium phthalimide (0.60 g, 3.3 mmol) in DMF (75 mL)

was stirred for 16 h at room temperature before the solution turned into yellow. The solvent was then evaporated in vacuo to give the residue. CH_2Cl_2 (100 mL) was used to extract the product which was washed with brine (100 mL), and the organic layer was dried with anhydrous Na_2SO_4. The solvent was evaporated in vacuo to give a solid crude product which was purified by column chromatography (silica gel, petroleum ether/ ethyl acetate 2:1) to give a yellow oil (1.40 g, 2.4 mmol, 80%).^{1}H NMR δ 7.82 (dd, 2H, J_1 = 5.4 Hz, J_2 = 3.1 Hz), 7.71 (dd, 2H, J_1 = 5.4 Hz, J_2 = 3.1 Hz), 6.89 (d, 2H, J = 8.5 Hz), 6.70 (d, 2H, J = 8.5 Hz), 6.01 (br, 1H), 4.24-4.09 (m, 3H), 3.97 (t, 2H, J = 5.8 Hz), 3.73 (t, 2H, J = 7.0 Hz), 3.65 (s, 1H), 3.45 (t, 2H, J = 5.1 Hz), 2.52 (d, 2H, J = 4.7 Hz), 2.17 (s, 3H), 2.16 (s, 3H), 1.88-1.78 (m, 4H). ^{13}C NMR δ 168.45, 168.22, 167.42, 157.11, 147.12, 145.97, 134.01, 132.01, 131.04, 123.19, 113.37, 101.44, 100.33, 67.00, 62.11, 60.40, 50.90, 50.09, 41.21, 37.65, 35.28, 26.49, 25.29, 19.14, 18.82. HRMS (FTMS ESI$^+$): m/z calcd for $C_{31}H_{33}N_5O_7Na^+$, 610.2278 $[M + H]^+$; found 610.2221.

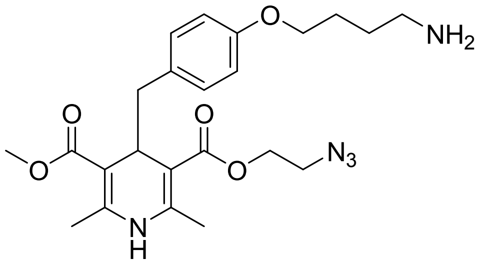

3-(2-Azidoethyl) 5-methyl 4-(4-(4-aminobutoxy)benzyl)-2,6-dimethyl-1,4-dihydropyridine-3,5-dicarboxylate (6)

A mixture of **5** (1.20 g, 21 mmol), hydrazinium hydroxide (0.5 mL) in ethanol (75 mL) was stirred for 10 h at room temperature. The solution was then basified with KOH solution (1 M, 50 mL), and extracted with CH_2Cl_2 (50 mL × 3) before the organic layer was dried with anhydrous Na_2SO_4. The solvent was evaporated in vacuo to give the residue (0.88 g, 92%) which was directly used in the next step without further purification.

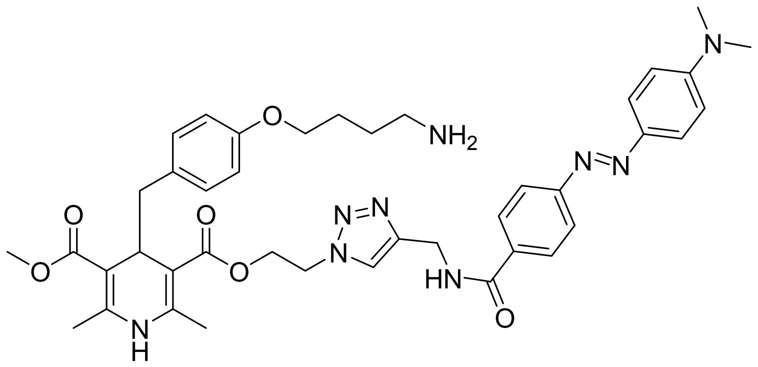

3-(2-(4-((4-((4-(Dimethylamino)phenyl)diazenyl)benzamido)methyl)-1H-1,2,3-triazol-1-yl)ethyl) 5-methyl 4-(4-(4-aminobutoxy)benzyl)-2,6-dimethyl-1,4-dihydropyridine-3,5-dicarboxylate (7)

A solution of **12** (0.82 g, 1.8 mmol), **6** (0.25 g, 1.8 mmol), CuI (0.015 g, 0.18 mmol) and N-ethyl-N- isopropylpropan-2-amine (DIPEA, 0.21 g, 3.6 mmol) in THF (50 mL) was stirred for 24 hours in room temperature before the solvent was evaporated in vacuo to give the residue which was purified by column chromatography (silica gel, $CH_2Cl_2/CH_3OH/Et_3N$ from 100:10:0 to 100:10:5) to give a red solid (0.84 g, 1.4 mmol, 79%). ^1H NMR δ 7.95-7.88 (m, 2H), 7.83-7.77 (m, 4H), 6.86-6.69 (m, 7H), 4.61-4.35 (m, 4H), 4.02-3.87 (m, 4H), 3.49-3.45 (m, 6H), 2.12-1.99 (m, 6H), 1.88-1.78 (m, 4H). ^{13}C NMR δ 167.91, 167.87, 157.19, 154.47, 153.31, 147.20, 143.10, 134.83, 131.08, 131.02, 129.84, 128.98, 128.89, 125.56, 121.95, 114.94, 114.47, 113.72, 112.04, 99.95, 67.77, 67.49, 60.55, 52.46, 50.91, 46.16, 41.65, 35.34, 33.74, 27.65, 27.12, 26.52, 19.34, 18.58, HRMS (FTMS ESI$^+$): m/z calcd for $C_{41}H_{50}N_9O_6^+$ 764.3884 $[M + H]^+$; found 764.3880.

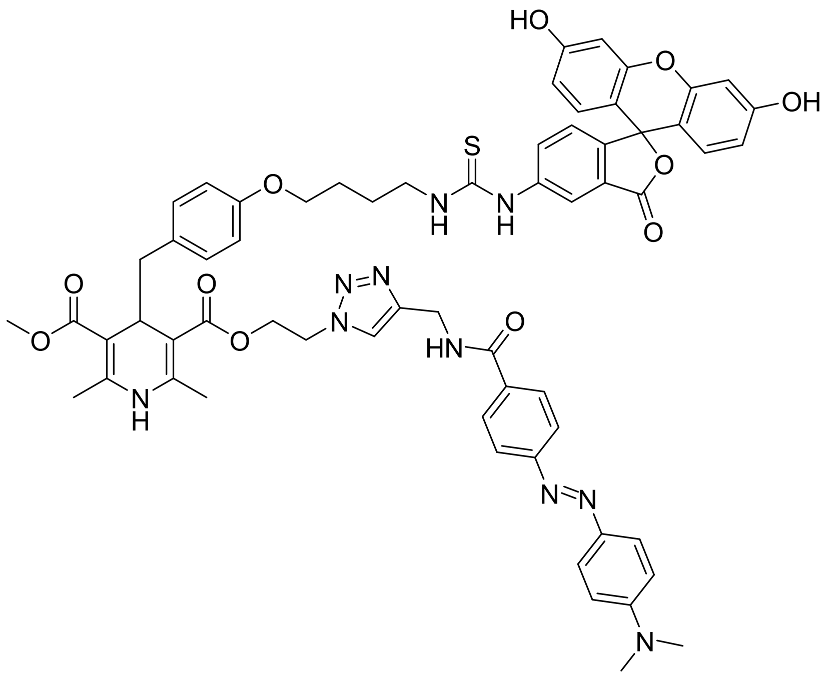

3-(2-(4-((4-((4-(Dimethylamino)phenyl)diazenyl)benzamido)methyl)-1H-1,2,3-triazol-1-yl)ethyl) 5-methyl 4-(4-(4-(3-(3',6'-dihydroxy-3-oxo-3H-spiro[isobenzofuran-1,9'-xanthene]-5-yl)thioureido)butoxy)benzyl)-2,6-dimethyl-1,4-dihydropyridine-3,5-dicarboxylate (8)

In a solution of **7** (202.4 mg, 0.26 mmol), fluorescein isothiocyanate (FITC, 123.6 mg, 0.31 mmol) Et_3N (3 mL) in DMF (50 mL) was stirred for 16 h. The solvent was evaporated in vacuo to give the residue which was purified by column chromatography (silica gel, CH2Cl2/CH_3OH 10:1) to yeild a red solid (113.8 mg, 0.10 mmol, 38%).^{1}H NMR δ 7.95-7.88 (m, 2H), 7.83-7.77 (m, 4H), 7.19-7.18 (m, 2H), 6.86-6.69 (m, 7H), 6.63-6.57 (m, 7H), 4.61-4.35 (m, 4H), 4.02-3.87 (m, 7H), 3.49-3.45 (m, 6H), 2.12-1.99 (m, 6H), 1.88-1.78 (m, 4H). ^{13}C NMR δ 191.79, 180.93, 179.72, 169.06, 168.82, 167.95, 167.91, 159.99, 159.93, 157.27, 157.23, 154.48, 153.30, 152.34, 152.28, 147.21, 143.07, 141.86, 134.77, 132.32, 131.09, 131.03, 130.99, 129.98, 129.54, 129.49, 128.96, 128.93, 128.87, 125.58, 121.95, 121.93, 115.39, 113.75, 113.62, 113.13, 113.06, 112.02, 111.95, 110.18, 109.92, 99.95, 68.27, 67.56, 50.94, 44.00, 41.62, 40.39, 35.34, 31.14, 30.02, 26.74, 26.71, 26.45, 25.65, 18.57, 18.39, 18.31, 13.13.. HRMS (FTMS ESI-): m/z calcd for $C_{62}H_{59}N_{10}O_{11}S^-$, 1151.4085 [M - H]$^-$; found 1151.4114.

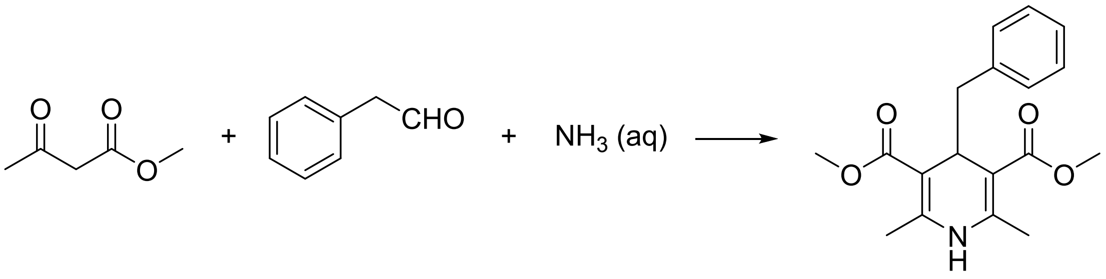

S

Scheme S2. Synthetic route of compound **S**.

Dimethyl 4-benzyl-2,6-dimethyl-1,4-dihydropyridine-3,5-dicarboxylate (Compound S)

A mixed solution of 2-phenylacetaldehyde (1.20 g, 10 mmol), methyl 3-oxobutanoate (2.32 g, 20 mmol) and 2 mL ammonia in 50 mL ethanol was stirred for 2.5 h under reflux condition. The solvent was evaporated in vacuo to give the residue which was then purified by column chromatography (silica gel, petroleum ether/ ethyl acetate 7:3) to give a pale yellow solid (1.95 g, 62%) ^{1}H-NMR ($CDCl_3$, 400 MHz) δ: 7.19-7.15 (3H, m), 7.39 (2H, dd, J_1 = 8.0 Hz, J_2 = 1.4 Hz), 5.34 (1H, s), 4.20 (1H, t, J = 5.6 Hz), 3.62 (6H, s), 2.56 (2H, d, J = 5.6 Hz) 2.19 (6H, s).

Specificity assay on Compound S in aqueous solution

1.0 mg compound **S** was dissolve in CH_3CN (20%) and H_2O (80%) to prepare a solution with a concentration of 0.0033 mM in a glass vial. 0.5 mM (150 equiv.) of O_2, H_2O_2, NO_3^-, NO_2^- or $OONO^-$ (suspicious reagents with dihydropyridine derivatives) were then bubbled or added into the mixture, respectively. Those vails were kept under 37 ^{o}C for 1 h, allowing enough time for any reaction. HPLC was used for measuring the decomposition rate of compound **S**. The measurement was completed in triplicate.

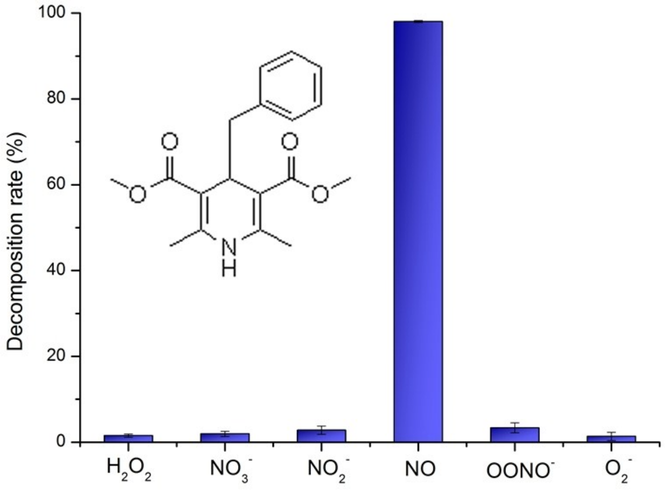

Figure S1. Compound S was exposed to 0.5 mM suspicious ROS/RNS. About 98% of compound S was found to be decomposed when treated with NO.

Correlation between NO amount and fluorescence intensity

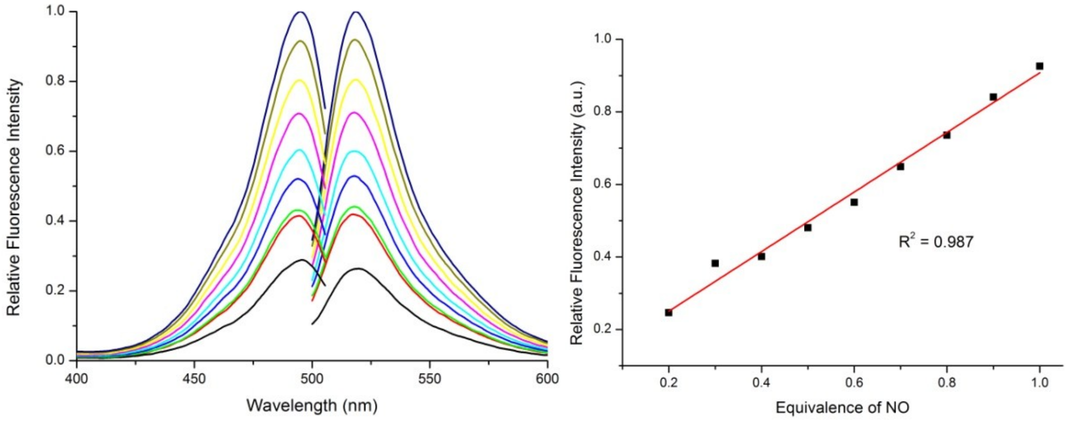

Figure S2. Fluorescence response of 10 μM DHPFQ to 1 equivalence NO in CH_3CN / PBS buffer = 1/4 (pH = 7.4, λ_{ex} = 490 nm, λ_{em} = 525 nm).

UV spectra assay

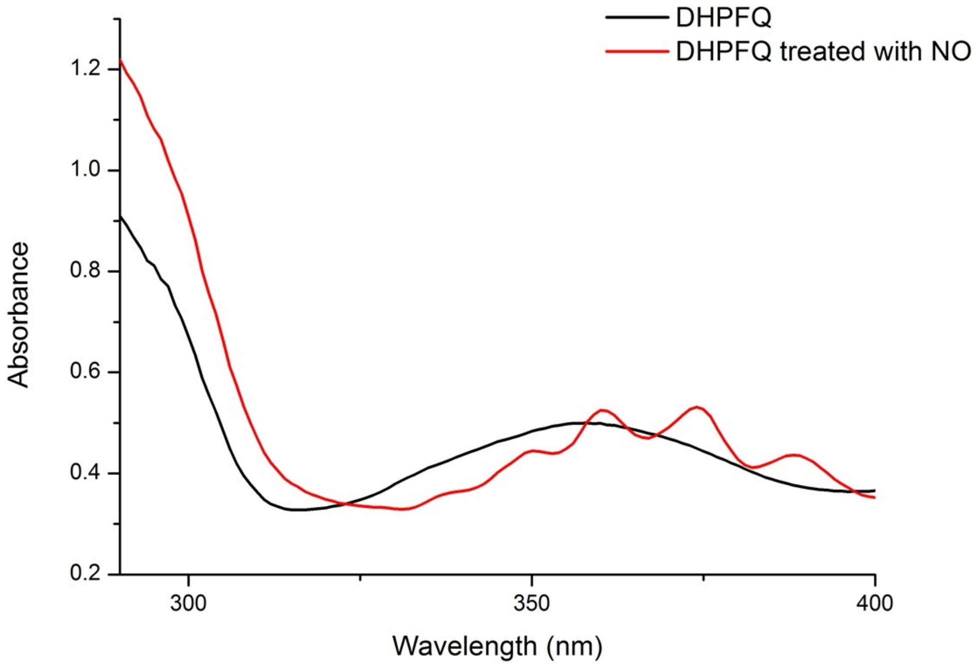

Figure S3. UV spectra of 10 μM DHPFQ and DHPFQ treated with NO in CH_3CN / PBS buffer = 1/4

Identification of the products of DHPFQ treated with NO

DHPFQ (1.0 mg) was dissolved in CH_3CN (0.2 mL) and H_2O (0.8 mL) and treated with excess NO. The solution was extracted by CH_2Cl_2 (1 mL) before the organic phase was evaporated in vacuo and sent to LC-MS determination immediately.

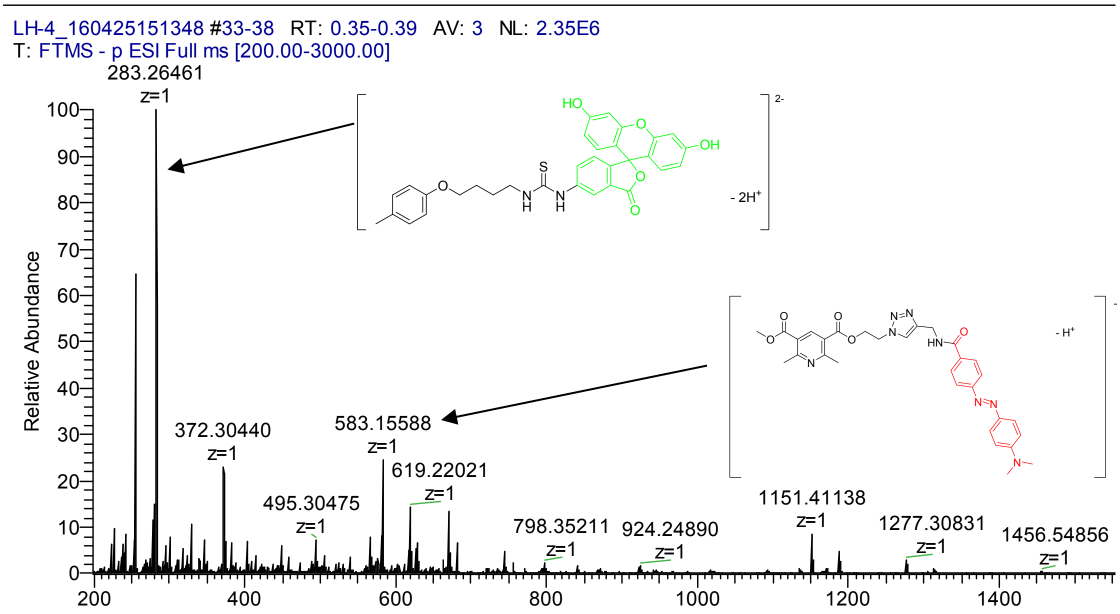

Figure S4. LC-MS of DHPFQ treated with NO

Cytotoxicity of DHPFQ

Cells were cultured continuously in DMEM High Glucose (DMEM; Themo Scientific) supplemented with 10 % fetal bovine serum (FBS; GIBCO), and antibiotics (penicillin 100 IU/mL, streptomycin 100 μg/mL) at 37℃ in a humidified atmosphere containing 5 % CO_2 . Twenty-four hours before the experiment,cells were grown in 96-well plates at an initial density 2 * 10^5 cells per well. The μM of probe were incubated for 48h then experiment were assayed using standard protocol with LDH Cytotoxicity Assay Kit (Beyotime Biotechnology Co,Ltd).

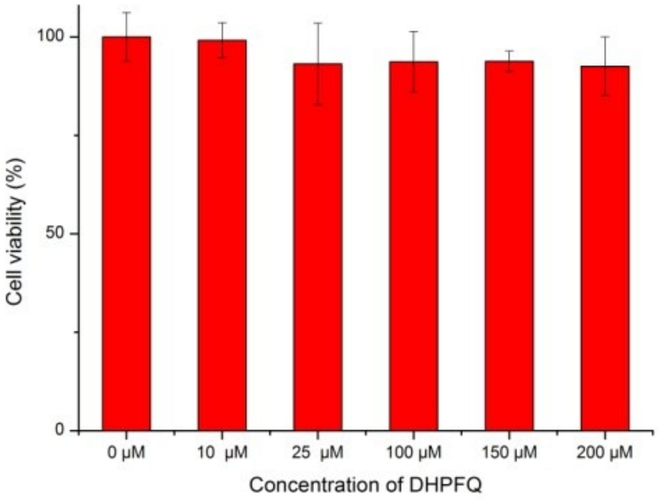

Figure S5. The viability of Raw 264.7 cells treated with DHPFQ

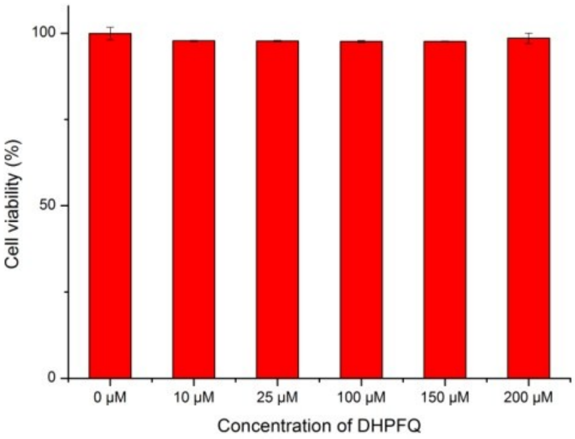

Figure S6. The viability of HeLa cells treated with DHPFQ.

Fluorescence imaging on fluorescence microscope

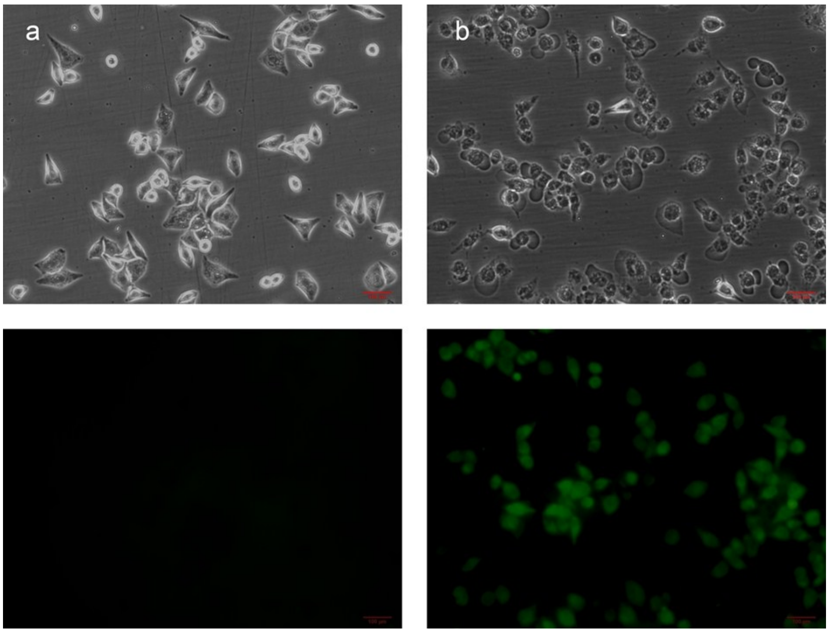

Figure S7. DHPFQ detection of NO in HeLa cells, bright field image (top) and corresponding fluorescence image (bottom). (a) Incubated with DHPFQ (50 μM) for 120 min; (b) co-treated with SNP (2 mM) and DHPFQ (50 μM) for 120 min. Scale bars are all 100 μm.

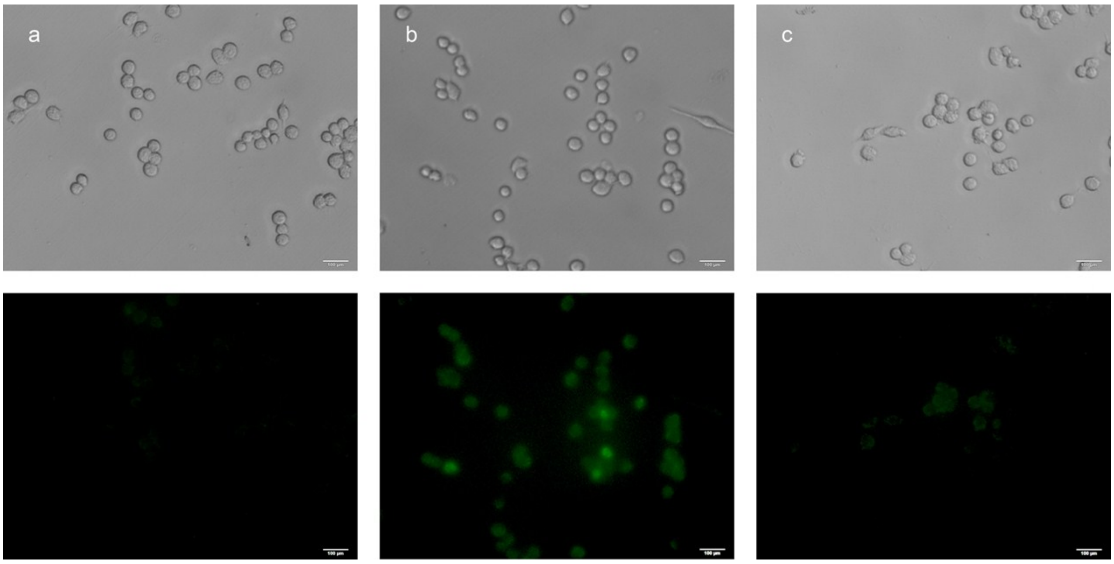

Figure S8. DHPFQ detection of NO in Raw 264.7 cells, bright field image (top) and corresponding fluorescence image (bottom). (a) Incubated with DHPFQ (50 μM) for 8 h; (b) pretreated with LPS (0.5 μg/mL) for 4 h and incubated with DHPFQ (50 μM) for 8 h; (c)

pretreated with N^G-monomethyl-L-arginine (2 mM), a NOS inhibitor, for 1.5 h and incubated with LPS (0.5 μg/mL) for 4 h, then DHPFQ (50 μM) for 8 h. Scale bars are all 100 μm..

Animal models

The normal ICR mice (five weeks, male, 20 g) were used for creating the arthritis models. Complete Freund's adjuvant (100 μL) was injected into the left hind metatarsal footpad by intradermal way. After two weeks from the first injection, another portion of complete Freund's adjuvant was injected at the same place. The *in vivo* imaging was acquired at 3 days after the second injection.

Immunohistology staining

The muscle tissues (normal region) and inflamed tissues (inflamed tissue) were collected and snap-frozen in optimal cutting temperature compound (OCT) at -80 ^oC. The tissues were sectioned into 10 μm slices for DAPI and F4/80 staining.

In vivo fluorescence imaging

To reduce the background absorption, depilatory paste was used to remove partial hair of mice. 470 nm and 600 nm were selected to be the exciting and emission wavelength. To raise the signal-noise-ratio, the exposure time was 5 min.

Appendix

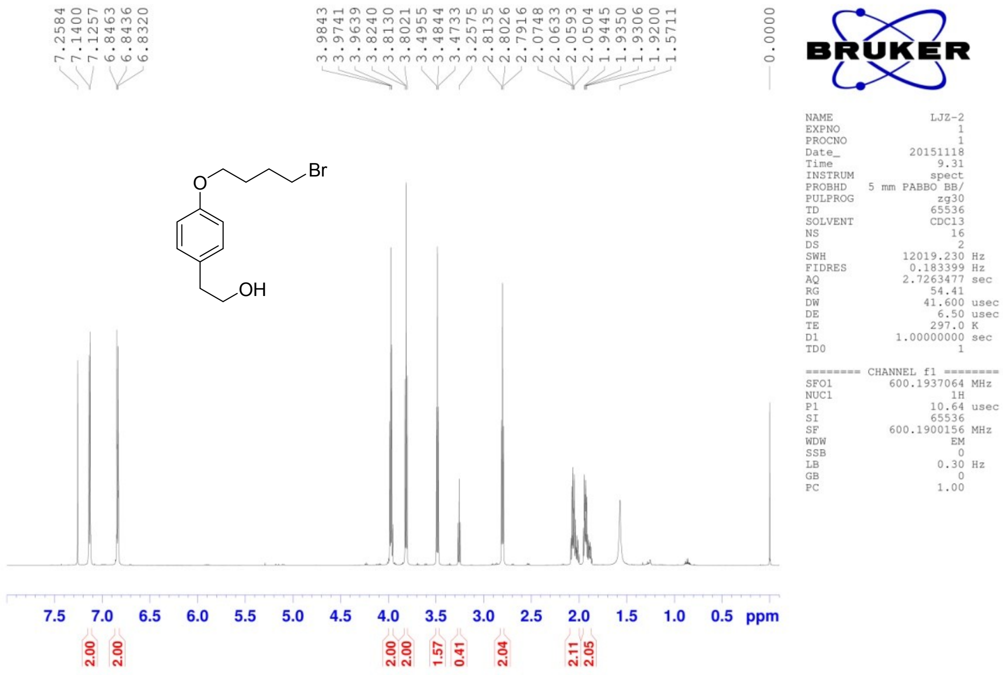

Figure S9. ^{1}H NMR of compound **9**

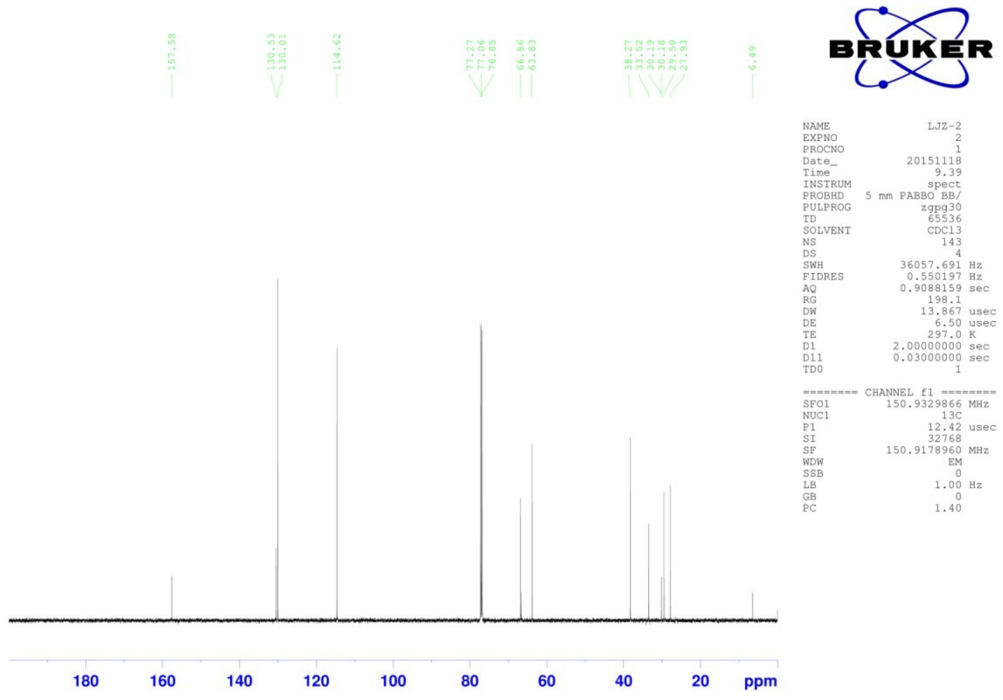

Figure S10. ^{13}C NMR of compound **9**

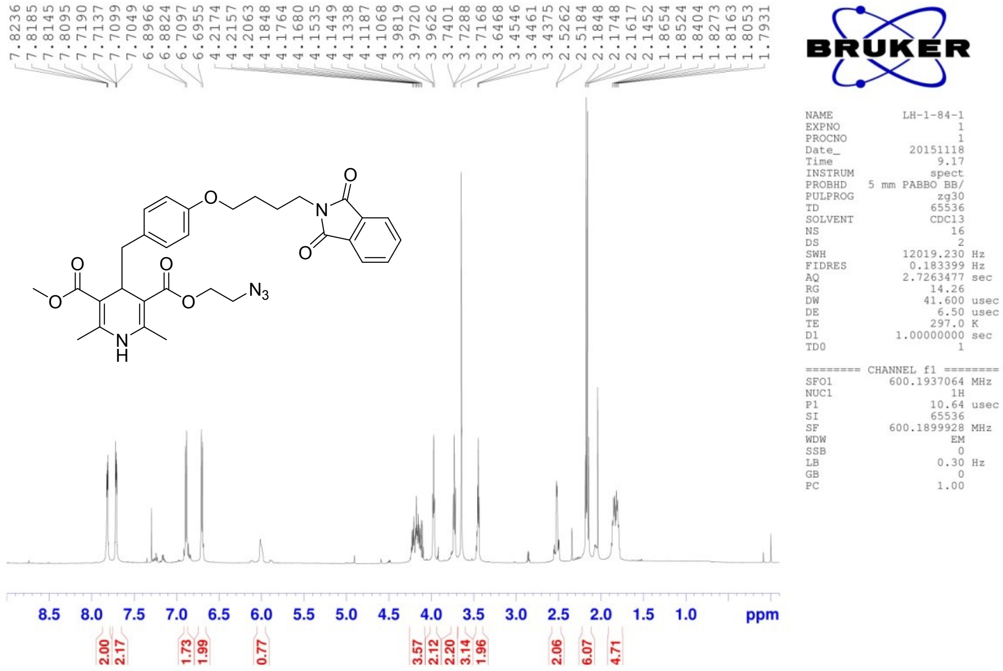

Figure S11. ^{1}H NMR of compound **5**

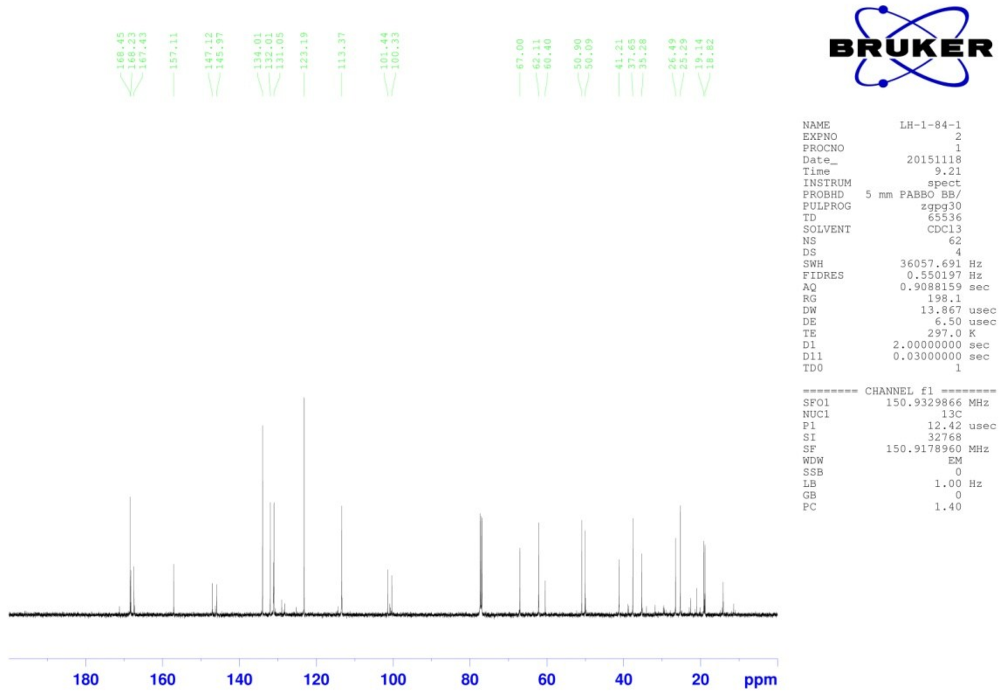

Figure S12. ^{13}C NMR of compound **5**

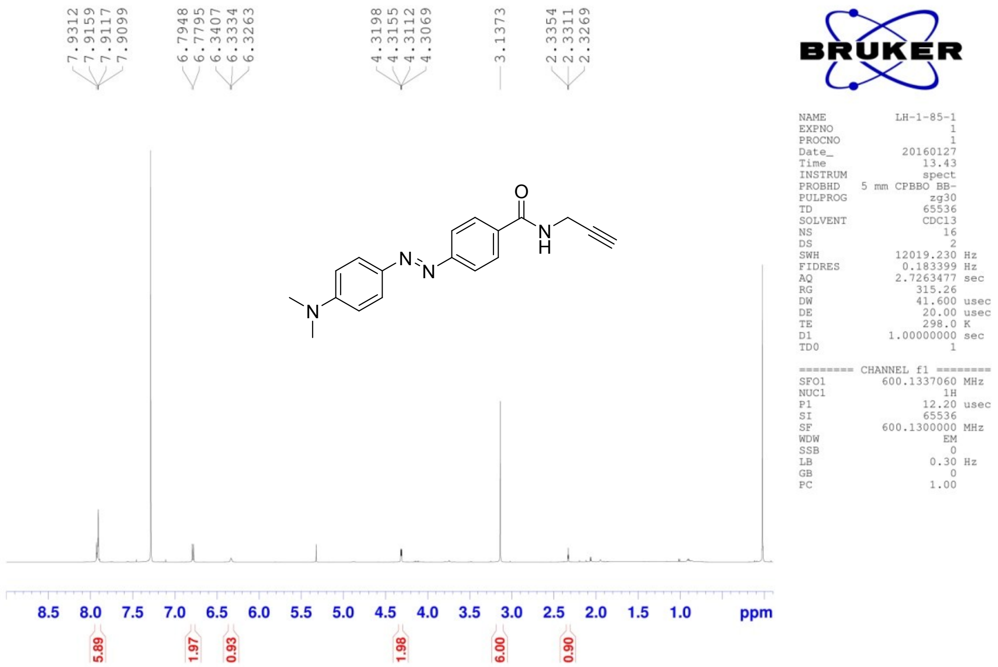

Figure S13. ^{1}H NMR of compound **12**

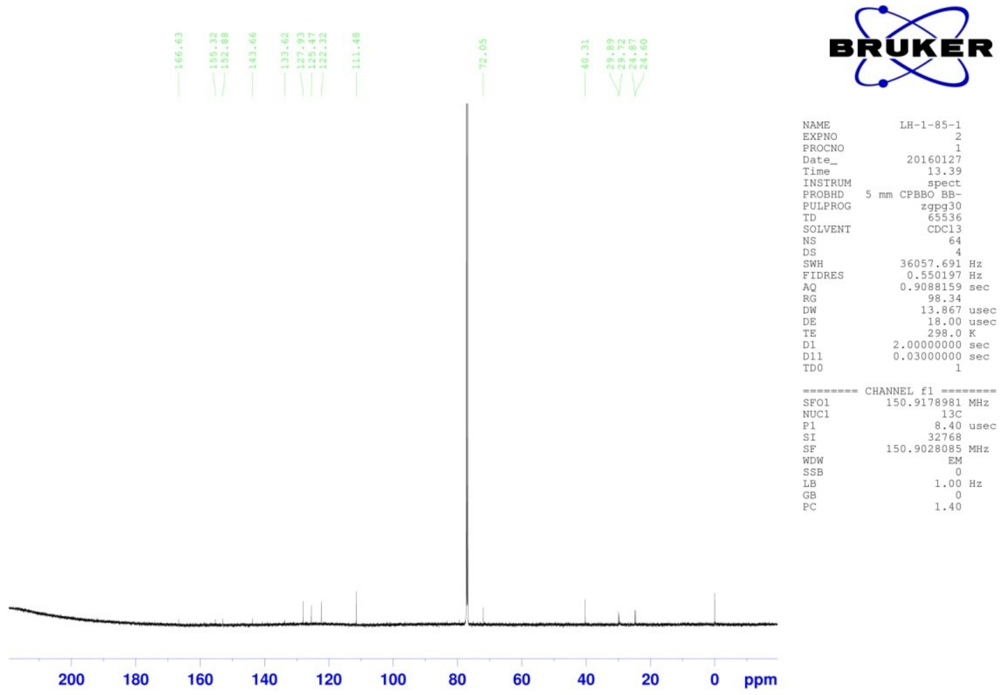

Figure S14. ^{13}C NMR of compound **12**

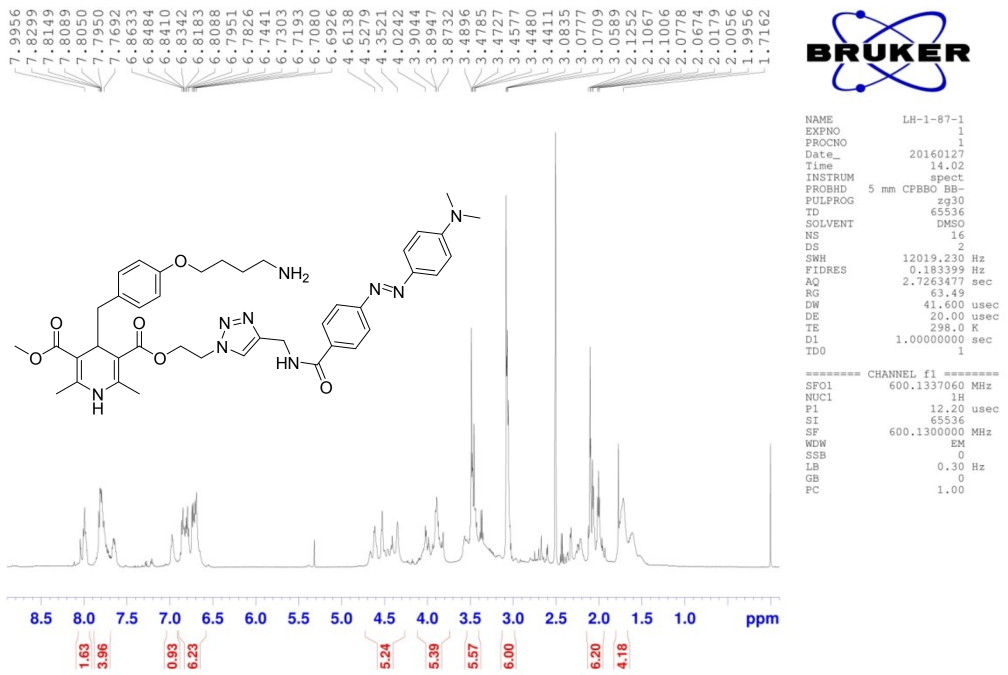

Figure S15. ^{1}H NMR of compound **7**

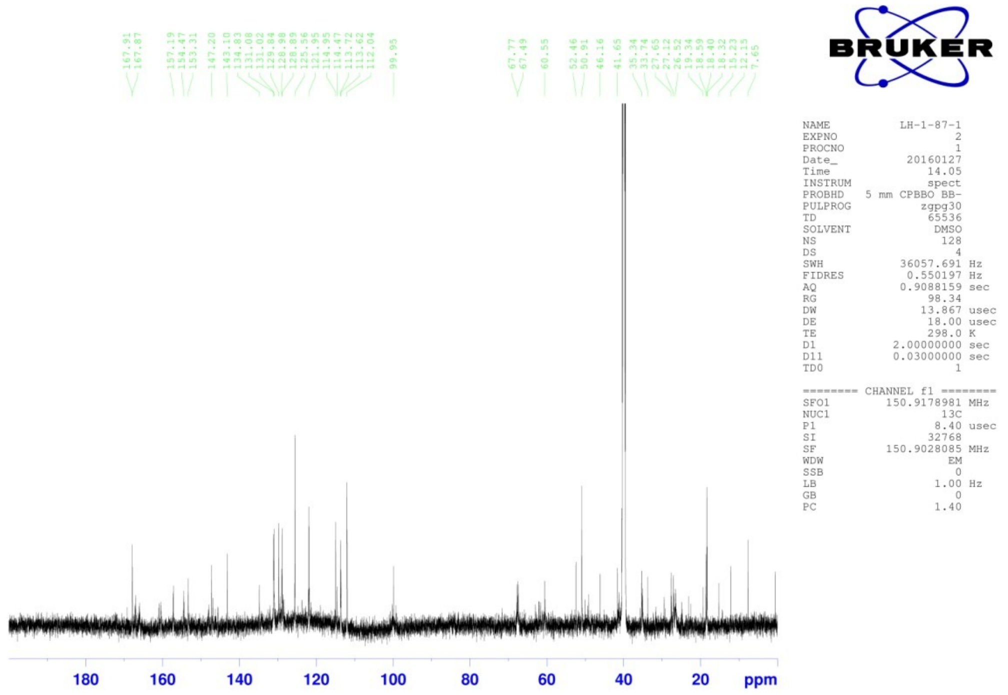

Figure S16. ^{13}C NMR of compound **7**

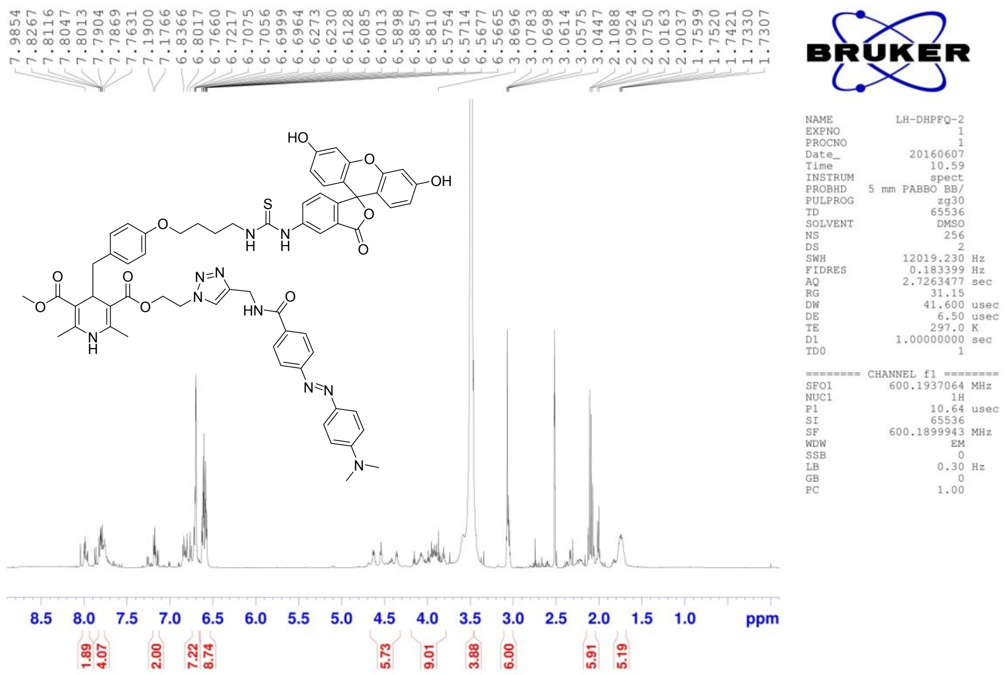

Figure S17. ^{1}H NMR of compound **8**

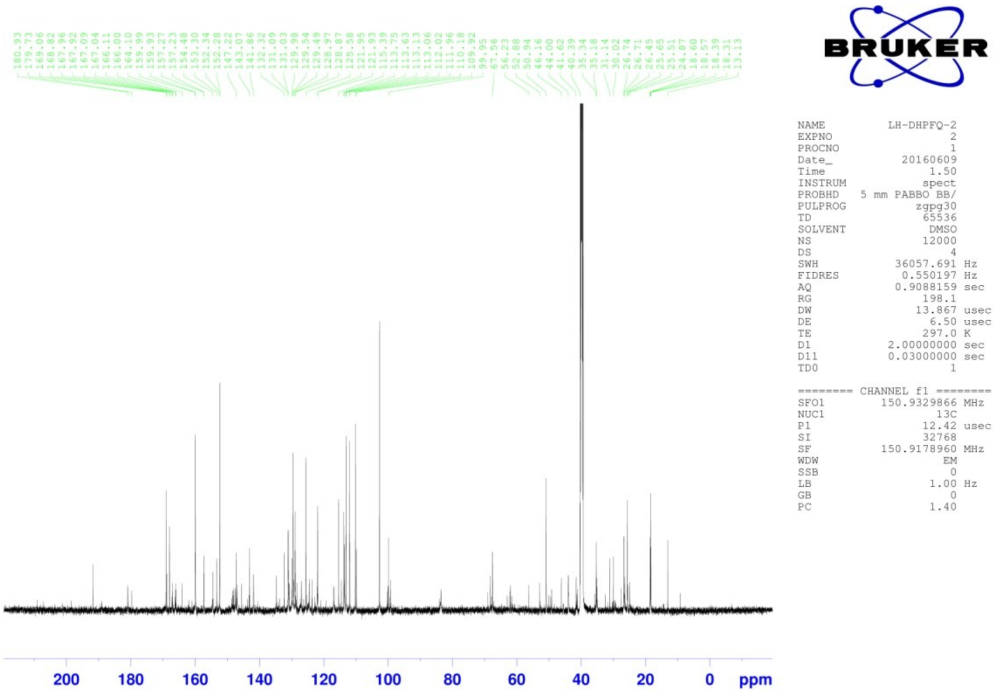

Figure S18. ^{13}C NMR of compound **8**

References

[1] N. Iqbal, E. E. Knaus, *Journal of heterocyclic chemistry* **1996**, *33*, 157-160;

[2] Y. Zhou, T. Kijima, S. Kuwahara, M. Watanabe, T. Izumi, *Tetrahedron Letters* **2008**, *49*, 3757-3761;

[3] Y. L. Jiang, C. A. McGoldrick, D. Yin, J. Zhao, V. Patel, M. F. Brannon, J. W. Lightner, K. Krishnan, W. L. Stone, *Bioorganic & medicinal chemistry letters* **2012**, *22*, 3632-3638.
